# Cellular senescence in naevi and immortalisation in melanoma: a role for p16?

**DOI:** 10.1038/sj.bjc.6603283

**Published:** 2006-08-01

**Authors:** V C Gray-Schopfer, S C Cheong, H Chong, J Chow, T Moss, Z A Abdel-Malek, R Marais, D Wynford-Thomas, D C Bennett

**Affiliations:** 1 Division of Basic Medical Sciences, St George's, University of London, Cranmer Terrace, SW17 0RE, UK; 2 The Institute for Cancer Research, London SW3 6JB, UK; 3 Division of Cellular and Molecular Medicine, St George's, University of London, London SW17 0RE, UK; 4 Department of Plastic Surgery, St George's Hospital, London SW17 0QT, UK; 5 Department of Dermatology, University of Cincinnati, Cincinnati, OH 45267, USA; 6 Department of Pathology, University of Wales College of Medicine, Cardiff, UK

**Keywords:** cellular senescence, immortalisation, melanoma, naevus, p16, p53

## Abstract

Cellular senescence, the irreversible proliferative arrest seen in somatic cells after a limited number of divisions, is considered a crucial barrier to cancer, but direct evidence for this *in vivo* was lacking until recently. The best-known form of human cell senescence is attributed to telomere shortening and a DNA-damage response through p53 and p21. There is also a more rapid form of senescence, dependent on the p16-retinoblastoma pathway. p16 (*CDKN2A*) is a known melanoma susceptibility gene. Here, we use retrovirally mediated gene transfer to confirm that the normal form of senescence in cultured human melanocytes involves p16, since disruption of the p16/retinoblastoma pathway is required as well as telomerase activation for immortalisation. Expression (immunostaining) patterns of senescence mediators and markers in melanocytic lesions provide strong evidence that cell senescence occurs in benign melanocytic naevi (moles) *in vivo* and does not involve p53 or p21 upregulation, although p16 is widely expressed. In comparison, dysplastic naevi and early (radial growth-phase, RGP) melanomas show less p16 and some p53 and p21 immunostaining. All RGP melanomas expressed p21, suggesting areas of p53-mediated senescence, while most areas of advanced (vertical growth-phase) melanomas lacked both p16 and p21, implying escape from both forms of senescence (immortalisation). Moreover, nuclear p16 but not p21 expression can be induced in human melanocytes by oncogenic *BRAF*, as found in around 80% of naevi. We conclude that cell senescence can form a barrier to melanoma development. This also provides a potential explanation of why p16 is a melanoma suppressor gene.

Cellular senescence, the growth arrest seen in normal mammalian cells after a limited number of divisions, is controlled by key tumour suppressors including p53 and p16, and is believed to be a crucial barrier to tumour development (Sharpless and DePinho, 2004). However, evidence for this overall concept comes very largely from cell cultures and animal models (Wynford-Thomas, 1999; Sherr, 2001; Sharpless and DePinho, 2004). No direct evidence has yet been presented that cell senescence forms a true barrier to development of human cancers *in vivo*, although improved evidence from animal models has emerged recently (Braig *et al*, 2005; Collado *et al*, 2005).

Two main pathways effecting normal cellular senescence in culture have been reported. The best known form of senescence, first described in fibroblasts, is effected primarily by the p53 pathway (Wynford-Thomas, 1999; Sherr, 2001; Sharpless and DePinho, 2004), and can be overcome by expression of exogenous hTERT (human telomerase reverse transcriptase, required for telomere maintenance), to give immortal (indefinitely proliferating) cultures (Bodnar *et al*, 1998). Here, senescence appears to be dependent on limited shortening of telomeres, activating p53 through checkpoint kinases 2 and possibly 1 (CHK2 and CHK1) (Stewart *et al*, 2003; d'Adda di Fagagna *et al*, 2003; Von Zglinicki *et al*, 2005). An alternative route to senescence was reported in certain epithelial cells. It involves only the p16/RB1 pathway, and the cells require interference with this pathway as well as hTERT expression, for immortalisation (Kiyono *et al*, 1998). p16-dependent senescence was abolished by disruption of the RB1 pathway, after which a further, p53-dependent growth arrest was observed. p16-dependent arrest of epithelial cells was, however, circumvented by culture with growth-inactivated fibroblast ‘feeder’ cells. The cells showed extended proliferation, p53-dependent senescence, and immortalisation with hTERT alone (Ramirez *et al*, 2001). The authors suggested that p16-dependent senescence was an artefact of imperfect culture conditions, leading to ‘stress’ (Ramirez *et al*, 2001).

In p16-dependent senescence, the cyclin-dependent kinase (CDK) inhibitor p16 is synthesised and activates RB1, by inhibiting phosphorylation of RB1 by CDK4 or CDK6 (Kiyono *et al*, 1998; Sharpless and DePinho, 2004). p16 is itself a tumour suppressor; germline mutations at its locus *CDKN2A* are principally associated with familial melanoma in humans, with some increased incidence of pancreatic cancer, suggesting particular importance in melanocytes (Gruis *et al*, 1995; Bennett, 2003; Hayward, 2003; Kefford *et al*, 2004; Gray-Schopfer and Bennett, 2006). In sporadic cancers, p16 alterations and deletions are more broadly distributed, being found in many types of cancer. The COSMIC database of over 13 000 cancer samples (Forbes *et al*, 2006) (http://www.sanger.ac.uk/genetics/CGP/cosmic/) lists p16 point mutations in 15% of all tested samples, including nearly every cancer type tested, although the frequency varies with type and culture status (22% for melanoma, 9% for all uncultured melanoma samples). This does not include deletions, and a recent study by comparative genome hybridisation highlighted p16 as the most commonly deleted gene in uncultured primary melanomas, at 50% frequency (Curtin *et al*, 2005). Thus, at least 59% of uncultured melanomas apparently have a p16 deficiency. To this can be added gene silencing; *CDKN2A* promoter methylation was reported in 19% of invasive primary melanomas (Straume *et al*, 2002). Moreover, a study with melanoma cell lines found that 100% of lines with normal p16 had a different alteration in the RB pathway (Bartkova *et al*, 1996). It is not clear how many other cancer types show such high total frequencies of somatic p16 alteration; but even if the specific relation between p16 and melanoma is restricted to germline mutations, it is interesting that there is evidence for the p16-dependent form of senescence in human melanocytes, even in a rich culture medium and with feeder cells (Bennett and Medrano, 2002; Sviderskaya *et al*, 2003). Two human melanocyte strains lacking functional p16 both showed a greatly extended lifespan, p53-dependent senescence, and immortalisation by hTERT alone (Sviderskaya *et al*, 2003).

These findings suggested a parallel with melanocytic lesions. Humans carrying p16 mutations have not only increased susceptibility to melanoma, but usually also numerous melanocytic naevi (moles), often large (Gruis *et al*, 1995; Bennett and Medrano, 2002; Bennett, 2003), implying a role for p16 in limiting naevus growth in these families. This led to surmises that moles may be melanocyte clones that have proliferated following a first mutation, then senesced (Bennett and Medrano, 2002; Mooi and Peeper, 2002; Bastian, 2003). Reports of activating *BRAF* mutations in most melanomas (Davies *et al*, 2002) and around 80% of naevi (Pollock *et al*, 2003) provided a likely candidate for the mutation that most commonly initiates proliferation. BRAF is a protein serine/threonine kinase that transduces mitogenic and other signals from RAS to MAPK (Robinson and Cobb, 1997) and can regulate cell proliferation. The most common oncogenic *BRAF* mutation in melanomas, V600E (formerly called V599E), results in constitutive ERK signalling *in vivo* and can transform immortal fibroblasts (Davies *et al*, 2002) and immortal mouse melanocytes (Wellbrock *et al*, 2004).

The senescence studies also suggested mechanisms for progression to melanoma. Malignant melanomas can originate in naevi, sometimes through intermediate lesions (Clark *et al*, 1984; Herlyn *et al*, 1985; Mooi, 1997; Hussein and Wood, 2002), as follows. Dysplastic naevi tend to be large, with architectural irregularities but not progressive growth, unlike melanoma (Mooi, 1997). Radial growth phase (RGP) melanomas are thin, growing only in or near the epidermis, while vertical growth phase (VGP) melanomas invade more deeply and are competent for metastasis and immortal in cell culture (Clark *et al*, 1984; Herlyn *et al*, 1985; Clark, 1991). We proposed a genetic model, in which benign and dysplastic naevi represent p16 and p53-dependent senescence, while melanomas arise by immortalisation of melanocytes (Bennett, 2003; Sviderskaya *et al*, 2003; Bennett, 2006). Published biological and molecular data (Talve *et al*, 1997; Keller-Melchior *et al*, 1998; Glaessl *et al*, 1999; Rudolph *et al*, 2000; Hussein and Wood, 2002; Bennett, 2003; Sviderskaya *et al*, 2003) were consistent with this model, but not conclusive, although a convincing report of cell senescence in melanocytic naevi has appeared recently (Michaloglou *et al*, 2005). We have now identified the genetic requirements for immortalisation of human melanocytes, and systematically studied molecular markers and mediators of cell senescence in clinical pigmented lesions of increasing malignancy. The results confirm that normal melanocyte senescence in culture is p16-dependent, show that the senescence in benign pigmented naevi is not p53-dependent but involves p16 expression, and support a key role for cell senescence in melanoma suppression. We also confirm and extend another finding of Michaloglou *et al* (2005) on senescence-like inhibition of normal melanocyte growth by exogenous ^*V600E*^BRAF. This inhibition correlates with upregulation of p16, but not p21.

## MATERIALS AND METHODS

### Melanocyte culture and gene transfer

Primary human melanocyte strains Nohm1 (Bennett *et al*, 1985), 830c (Scott *et al*, 2002) and HM303CN (Minwalla *et al*, 2001) were grown as described (Bennett *et al*, 1985), except in the following melanocyte medium (Sviderskaya *et al*, 2003): RPMI 1640 medium, 10% foetal calf serum, 200 nM 12-*O*-tetradecanoyl phorbol 13-acetate (Sigma Chemical Co., Poole, UK), 200 pM cholera toxin (Sigma), 10 ng ml^−1^ human stem cell factor (R&D Systems, Abingdon, UK) and 10 nM endothelin 1 (Sigma), gassed with 10% CO_2_. Supplements shortly after retroviral infection also included 1 *μ*M insulin, 40 pM fibroblast growth factor 2 (R&D Systems, Abingdon, UK) and 1 *μ*g ml^−1^ *α*-tocopherol. Melanocytes for the BRAF studies only were obtained from Cascade Biologics and were grown in medium 254 with HMGS supplements (Cascade Biologics, Mansfield, UK). WM266.4 melanoma cells (ATCC), all producer cells and HeLa cells were grown in DMEM with 10% calf serum. Retroviral gene transfer into melanocytes was largely as described (Sviderskaya *et al*, 2003). All vectors, with or without inserts, were from Dr CJ Jones (Pathology, University of Wales College of Medicine, Cardiff, UK); the HPV16-E7 vector was originally from D Galloway (Fred Hutchinson Cancer Research Center, Seattle, WA, USA). pBABEpuro or pBABEneo vectors were transfected into the Omega E ecotropic packaging line, and supernatant containing virus used to infect *φ*CRIP amphotropic packaging cells (Danos and Mulligan, 1988). LXSN vectors were grown in PA317 amphotropic packaging cells (Halbert *et al*, 1991). *φ*CRIP or PA317 medium containing infectious virus was harvested (using appropriate containment procedures) and frozen. Melanocytes were infected 1 day after plating them at 4–5 × 104 ml^−1^. Supernatant was thawed, filtered (0.45 *μ*m), supplemented with polybrene (3.5 *μ*g ml^−1^), and incubated overnight with melanocytes. Conditioned medium from the melanocytes before infection was then replaced. Normal culture was resumed after 2–3 days. For infection with a second vector, the procedure was repeated 2 weeks later. Inserts included hTERT cDNA (Geron Corp., Menlo Park, CA, USA) in pBABEpuro and HPV16-E7 in LXSN, as described before (Halbert *et al*, 1991; Sviderskaya *et al*, 2003). The p16 antisense vector contained p16 exon 1*α* cloned in the antisense orientation between the *Bam*H1 and *Eco*R1 sites of pBABEneo and expressed from the viral LTR. Normal human CDK4 cDNA was likewise expressed from the viral LTR of pBABEneo. Some melanocyte cultures following infection were plated on XB2 keratinocyte feeder cells (3 × 10^4^ ml^−1^) (Sviderskaya *et al*, 2003).

Transient transfection was by Nucleofector™ technology, using the manufacturer's protocols (Amaxa GmbH, Cologne, Germany). 5 × 10^5^ melanocytes were transfected with 5 *μ*g DNA of Myc-tagged pEFm vector containing either no insert, wild-type or human ^*V600E*^*BRAF* sequences, and cultured in 1 ml normal medium in a well of a six-well plate for 5 days before assays.

### Growth curves and calculation of population doublings

Two near-confluent cultures were each counted in triplicate by haemocytometer, pooled and replated at a recorded density. The relative population increase during that passage was calculated and converted to population doublings.

### Immunoblotting

Cells were washed twice in PBS, covered in RIPA lysis buffer (Sviderskaya *et al*, 2003) and scraped off the plates. Protein concentration was quantified using the bicinchoninic acid (BCA) method. Protein (30 *μ*g) were electrophoresed through a 12% SDS–polyacrylamide gel and transferred to PVDF membranes by semi-dry blotting. Immunodetection was by enhanced chemiluminescence (Amersham, Little Chalfont, UK). Antibodies used were mouse anti-p16 (Novocastra, Newcastle, UK, or from NeoMarkers, Newmarket, UK for the BRAF work); rabbit anti-Myc (Abcam, Cambridge, UK), rabbit anti-p21 and rabbit anti-ERK (Santa Cruz/Autogen Bioclear, Mile Elm, UK), and mouse anti-phospho-ERK (Sigma). Second antibodies were appropriate HRP-conjugated anti-mouse or rabbit Igs (Stratech, Soham, UK).

### Immunostaining of lesion sections

Formalin-fixed, paraffin-embedded clinical specimens were obtained from St George's Hospital with approval from the local Research Ethics Committee. JC or HC performed histopathological diagnosis. Adjacent, parallel sections from the same lesions were used for direct positional comparisons of staining for p16, Mart1, p53, p21 and CHK2. 4-*μ*m sections were mounted on poly-L-lysine-coated slides. Sections were deparaffinised, rehydrated in Histoclear and placed in deionised water for at least 30 s. Target retrieval was by microwave heating with 0.01 M citrate buffer, pH 6.0. Slides were rinsed with 50 mM TBS wash buffer with 0.3 M NaCl and 0.1% Tween. Immunohistochemistry used the EnVision Fast Red system with alkaline phosphatase (DakoCytomation, Ely, UK). Antibodies were at recommended dilutions. Anti-p16, p53 and MART-1 antibodies were from Novocastra, anti-CHK2 from Autogen Bioclear, and anti-p21 from BD Pharmingen, Oxford, UK. Several types of lesions including positive and negative control sections were always immunostained simultaneously.

### Immunostaining of cultured cells

Normal human neonatal epidermal melanocytes (Cascade Biologics, Mansfield, UK) and control cell lines were grown on glass coverslips. Cultures were fixed with fresh 3.7% formaldehyde in PBS. Permeabilisation was in 0.5% Triton X-100 in PBS. Nonspecific sites were blocked in 1% bovine serum albumin in PBS. The cells were exposed to anti-p16 (NeoMarkers), anti-Myc tag (Abcam), or anti-Ki67 (Abcam) primary antibodies followed by either anti-mouse Cy3 or anti-rabbit Cy2 secondary antibodies (Jackson ImmunoResearch Laboratories). Cells were counterstained with DAPI and mounted in DABCO (Sigma).

### Detection of acidic *β*-galactosidase (Dimri *et al*, 1995)

Frozen sections of benign congenital naevi of minimal diameter 10 mm were used. Biopsy specimens were mounted in OCT embedding compound and rapidly frozen in isopentane. Frozen sections were mounted on poly-lysine-coated slides, thawed, fixed (1 min) in 1% formaldehyde in PBS, and washed twice in PBS. They were immersed in staining solution (10% PBS, 3.95 M K_3_Fe(CN)_6_, 5 M K_4_Fe(CN)_6_, 1 M MgCl_2_, 1 mg ml^−1^ X-gal, pH 6.0) for 24 h at 37°C. Sections were rinsed, counterstained with eosin Y, rinsed, dehydrated, cleared and mounted. Senescent cultured melanocytes gave a positive control.

## RESULTS

### Immortalisation of normal human melanocytes

We previously reported that p16-deficient but not normal human melanocytes could be immortalised by retrovirally mediated expression of hTERT (Sviderskaya *et al*, 2003), suggesting that RB1-pathway deficiency and hTERT expression were sufficient for melanocyte immortalisation. However, this evidence was weakened by the possibility that other genetic aberrations might be present in the two p16-deficient melanocyte strains used (Sviderskaya *et al*, 2003), as these were from familial melanoma patients. We have now tested the immortalisation requirements of three normal human melanocyte strains. We used the following exogenous genes to disrupt p16/RB1 signalling: an antisense p16 sequence, CDK4 cDNA overexpressed from a viral promoter, and the E7 oncogene of human papillomavirus 16 (HPV16-E7). Melanocytes were infected with amphotropic retroviruses containing hTERT or no insert, and separately infected with an RB1-disrupting vector or a control vector. Some infected cultures were initially grown with keratinocyte feeder cells.

**Figure 1:**
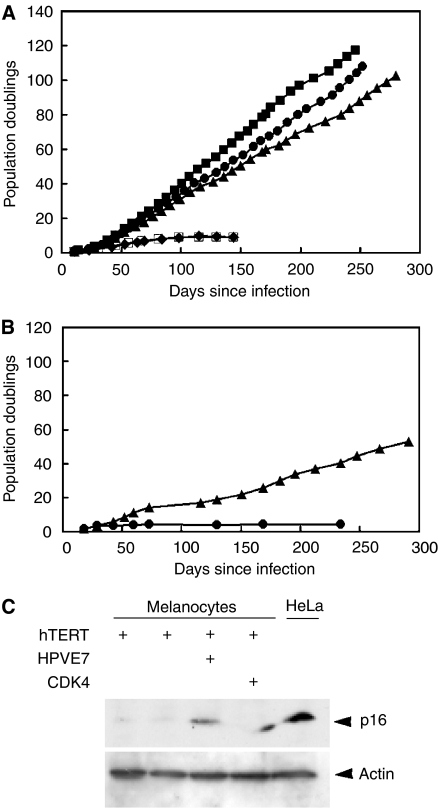
Genetic requirements for immortalisation of normal human melanocytes. (**A**) Growth of a representative human melanocyte strain (830c) following transduction of the indicated experimental or control sequences with pBABE amphotropic retroviruses. hTERT was added two weeks before the other sequences. Time since the second infection is shown. Immortalisation was seen following infection with hTERT in combination with each of the other experimental sequences. (▪) HPV16-E7+hTERT. (•) CDK4+hTERT. (▴) Antisense p16+hTERT. (□) pBABEpuro+pBABEneo. (⧫) pBABEpuro+pBABEpuro. Two other human melanocyte strains, Nohm-1 (Bennett *et al*, 1985) and HM303CN gave similar results (not shown). The resulting immortal melanocyte lines were generically called Hermes 3 (from Nohm-1), Hermes 4 (from 830c) and Hermes 5 (from HM303CN) (see (Sviderskaya *et al*, 2003) for Hermes 1 and 2). Growth was monitored until cells either stopped growing or achieved at least twice the number of population doublings for that cell strain's normal lifespan (full curve not necessarily shown), when they were deemed immortal. (**B**) Growth of 830c melanocytes following infection with hTERT only. In 2/6 cases (one shown here), immortalisation was seen after a lag, with hTERT only. (▴) hTERT; (•) pBABEpuro. (**C**) Immunoblot analysis for p16, showing negligible p16 expression in the two normal melanocyte cultures that immortalised with hTERT only. First lane: HM303CN melanocytes with hTERT. Second lane: 830c melanocytes with hTERT. 3rd and 4th lanes: Nohm-1 melanocytes with hTERT and with HPV16-E7 or CDK4 as indicated. HeLa: HeLa cells as positive control for p16 expression.

We previously reported that normal melanocytes could not be immortalised using hTERT alone, even in the presence of feeder cells (Sviderskaya *et al*, 2003). However, as shown in Figure 1A, hTERT in combination with any of three approaches to disrupt the p16/RB1 pathway could immortalise normal melanocytes. Similar results were obtained with all three independent normal melanocyte strains. Cytogenetic analysis showed initially normal diploid karyotypes for most of the immortalised cultures. Immunoblotting of cultures infected with antisense p16 and sense CDK4 confirmed repression of p16 expression and increased CDK4 expression, respectively (data not shown). hTERT alone once again gave no immortalisation in 4/6 experiments, including two cultures grown with feeder cells. Slowly growing immortal cells were obtained in 2/6 experiments, but in both cases progressive growth started only after a growth plateau and long lag of several months (Figure 1B), compared to immediate growth in the presence of CDK4, antisense p16 or HPV16-E7 as well as hTERT (Figure 1A). Moreover, immunoblot analysis (Figure 1C) revealed virtually no p16 in either of the two cultures that immortalised with hTERT alone. p16 is normally expressed by high-passage human melanocytes (Bandyopadhyay *et al*, 2001; Bennett and Medrano, 2002), and has thus been silenced in the cells that eventually grew in these two cultures, probably (given the long lag before growth) from a small number of variant cells. In comparison, the cells infected with HPV16-E7 and with overexpressed CDK4, where the entire culture grew immediately, did not lose p16 protein (Figure 1C). This is as expected, since these two proteins directly inhibit the p16/RB pathway, so there would be no selection for cells lacking p16. In summary, in 6/6 cases, hTERT expression failed to immortalise normal melanocytes without p16/RB pathway disruption, whereas with each of three gene combinations expressed in three melanocyte strains, p16/RB pathway disruption combined with hTERT did immortalise melanocytes efficiently and with no lag.

### Evidence of p16-dependent senescence in benign naevi

**Figure 2:**
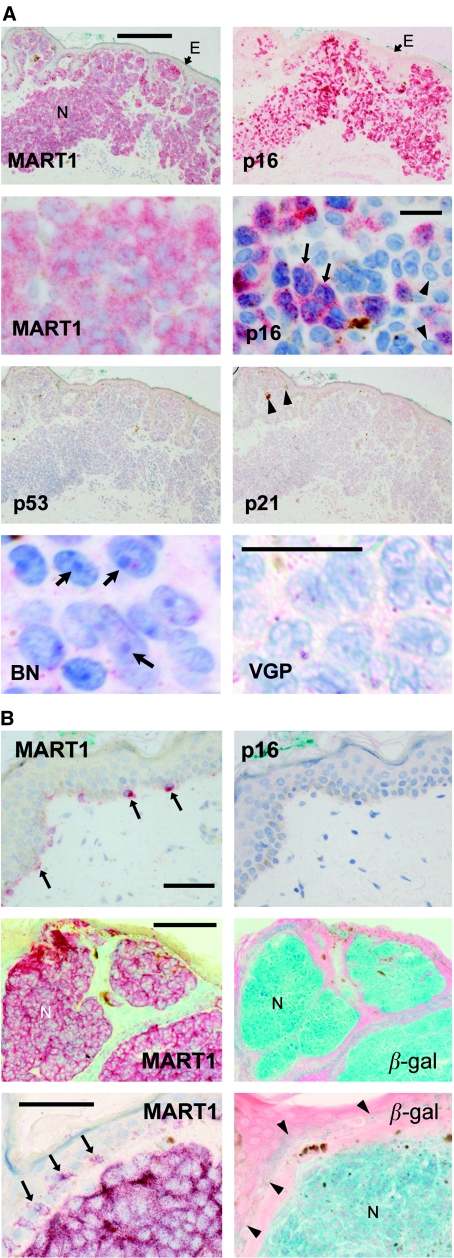
Typical expression of senescence-associated proteins in benign compound naevi and normal epidermis. Immunostained proteins appear red and nuclei blue (haematoxylin counterstain, except where noted). Acidic *β*-galactosidase gives a blue colour, with eosin (pink) counterstain. (**A**) Top and second rows, detection of melanocytic marker MART1 to show melanocytes and for p16 in parallel sections of the same biopsy. Top panels, low magnification of compound naevus (N), showing intense p16 immunostain (without haematoxylin). E, epidermis. Scale bar, 200 *μ*m. Second row, higher magnification of another naevus to show detail. Contrast slightly enhanced in right image to clarify chromatin detail. Scale bar, 20 *μ*m. Levels of p16 expression vary, with some cells not visibly positive. Immunostain where present is both nuclear and cytoplasmic. Arrows: large multinucleate cells; arrowheads: large nucleoli. Third row, parallel sections of same naevus as top panels, immunostained for p53 and p21 as indicated. No p53 or p21 was detected. Brown material (arrowheads) is melanin pigment. Bottom row, high magnification of haematoxylin-stained areas of a benign compound naevus (left), showing heterochromatic foci in nuclei (arrows), and of a VGP melanoma (right) showing absence of such foci. Note that lesions are heterogeneous and not all areas showed such clear differences. Contrast digitally enhanced as sections were lightly stained. Scale bar, 20 *μ*m. (**B**) Top, normal adjacent epidermis from same sections as (**A**) top. Scale bar, 50 *μ*m. MART1 (left) shows distribution of normal melanocytes (arrows), which have no detectable p16 (right). Middle and bottom, MART1 and acidic *β*-galactosidase in parallel sections of a benign congenital naevus. All cells of naevus (N) show both markers. Higher magnification (bottom) shows some acidic *β*-galactosidase reactivity in whole basal epidermis (arrowheads), but faint compared to naevus cells. Note: frozen sections were needed for this stain, and were available only for congenital naevi. Arrows, melanocytes. Scale bars, 150 *μ*m (middle), 50 *μ*m (bottom).

We now used immunostaining to test expression of markers and mediators of p16- and p53-dependent senescence in sections of benign and malignant melanocytic lesions. p16 was clearly detectable in 100% of benign compound naevi examined (Figure 2A, Table 1). Typically, staining was found throughout the lesion, although at the single-cell level, lesions were patchworks of immunostained and unstained naevus cells (Figure 2A). As illustrated, there was no detectable variation of p16 immunostaining with depth in these lesions. p16 was nearly always nuclear as well as cytoplasmic in naevi; nuclei stained with Fast Red as well as haematoxylin appear purple rather than blue (Figure 2A). Naevus cells often appeared large and sometimes multinucleate (Figure 2A), properties seen in senescent melanocytes (Bennett and Medrano, 2002). Prominent heterochromatic foci were often visible in the nuclei (Figure 2A, lower left), reminiscent of the heterochromatic foci described in senescent cells in culture (Narita *et al*, 2003). These foci appeared to be uncommon in melanomas, as illustrated for a VGP lesion (Figure 2A, lower right), although such morphological features are variable and difficult to quantitate. Importantly, no p16 was detected in normal epidermal melanocytes (Figure 2B). The region illustrated is typical of normal epidermis (well separated from the lesion), which we examined in all specimens where it was present, totalling at least several hundred melanocytes. No p16 immunostaining was found. Naevi were then stained for acidic *β*-galactosidase activity, or ‘senescence-associated *β*-galactosidase’ (Dimri *et al*, 1995; Sharpless and DePinho, 2004). This appears to be expressed reliably by senescent cells, although also by some other cells, including human cultured melanocytes (Dimri *et al*, 1995). It appears to be lysosomal *β*-galactosidase (Kurz *et al*, 2000). (This marker was studied only in large congenital naevi, since fresh, frozen sections were required and acquired naevi were too small for material to be spared from routine diagnosis for freezing.) We observed strong to moderate acidic *β*-galactosidase activity in 7/7 benign congenital naevi (Figure 2B). In comparison, there was no specific staining of normal epidermal melanocytes (Figure 2B).

Benign naevus cells thus did appear senescent, from two molecular markers and several biological and morphological properties (also including growth stasis). To address what type of senescence had occurred, naevi were immunostained for p53 and p21. p21 should be expressed in p53- but not p16-dependent senescence, while p53 levels are likely to be detectable in cells undergoing p53-dependent senescence (Sviderskaya *et al*, 2003). p21 was not detected in any of nine benign naevi, and only 1/7 showed traces of p53 (Table 1; Figure 2A). Thus the senescence in benign melanocytic naevi is not of the p53-dependent type, and so is either p16-dependent or some novel form of senescence.

### Evidence for escape from p16-dependent senescence in dysplastic naevi

**Figure 3:**
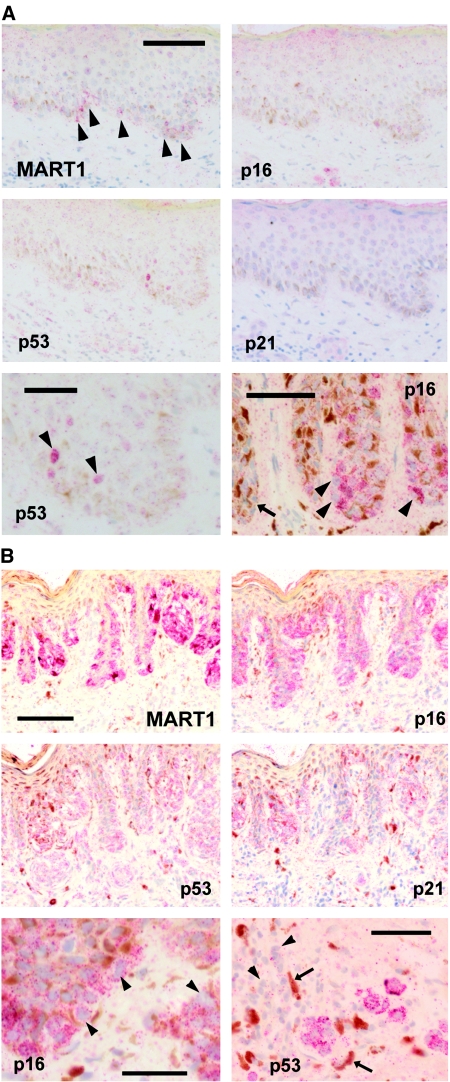
Immunostaining of senescence effectors in dysplastic naevi. (**A**) lesions from normal patients. Top and middle, MART1 shows naevus cells throughout basal epidermis in this lesion, yet there is little or no p16, little p53 and no p21 detected in parallel sections. Scale bar, 100 *μ*m. Bottom left, detail of p53 immunostaining in same lesion showing a few positive nuclei (arrowheads). Scale bar, 50 *μ*m. Bottom right, detail of p16 reaction in a different dysplastic naevus. Scale bar, 50 *μ*m. Most naevus cells here are strongly positive for p16, yet the reaction appears generally cytoplasmic (arrowheads: unstained nuclei). Arrow: other parts of this naevus had little or no p16. (**B**) lesion from a patient lacking active p16 because of two germline mutations. MART1 and p16 immunostaining (top) show similar patterns: nearly all naevus cells were prominently positive for p16. Many naevus cells are also positive for p53 and p21 (middle). Scale bar, 100 *μ*m. Bottom left: p16 reaction is cytoplasmic only (arrowheads: unstained nuclei). Scale bar, 30 *μ*m. Bottom right: p53-positive naevus cells in dermis, in same lesion, surrounded by pigmented macrophages (arrows) and lymphocytes (arrowheads). Scale bar, 50 *μ*m.

Dysplastic naevi also all expressed some p16, although reactivity was more patchy (Figure 3A) and half were predominantly negative (Table 1). Interestingly, p16 was detected only in the cytoplasm in some areas of these lesions (Figure 3A), whereas it generally appeared both nuclear and cytoplasmic in benign naevi (Figure 2A). Cytoplasmic p16 may sometimes be nonfunctional (see Discussion). In culture, p16 deficiency can lead to prolonged cell proliferation and p53-dependent senescence, as in cultured p16-deficient melanocytes (Sviderskaya *et al*, 2003). A proportion of dysplastic naevi did indeed show pockets of p53-positive cells, sometimes with detectable p21 (Table 1; Figure 3A). p53 location was nuclear or nuclear and cytoplasmic. Typically, however, neither protein was observed in most of the lesion.

### An atypical naevus from a p16-deficient patient: high p16 expression and possible p53-dependent senescence

Naevi from patients with biallelic p16 mutations tend to be large. We surmised that these might exhibit p53-dependent senescence, as confirmed for cultured melanocytes from these patients (Sviderskaya *et al*, 2003). We analysed an atypical naevus from one of the same two patients with biallelic point mutations in p16 (Huot *et al*, 2002) (Figure 3B). The mutant p16 was strongly expressed in nearly all lesional cells, and not in normal adjacent skin. This p16 protein was mostly cytoplasmic, however (Figure 3B), consistent with its known dysfunctionality in this patient (Huot *et al*, 2002). p21 and p53 were indeed expressed widely in this naevus, generally together, although less ubiquitously than p16 (Figure 3B). Pigmented melanophages (macrophages that have ingested melanin) and lymphocytes were often found near dermal p53-positive cells (Figure 3B), suggestive of melanocyte death (see Discussion).

### Further loss of p16 and p21 expression with melanoma progression

**Figure 4:**
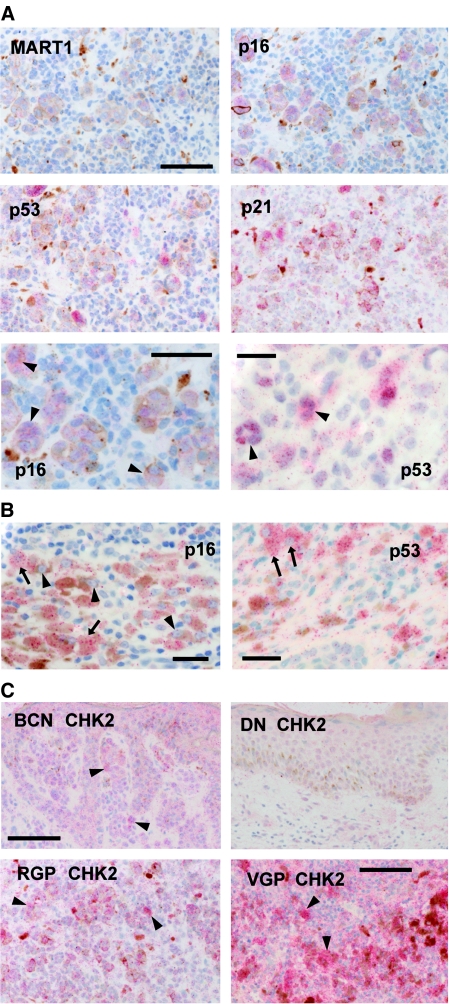
Typical immunostaining of senescence mediators in melanomas and naevi. (**A**) RGP melanomas. Top and middle: The same area of one melanoma is positive for MART1, p16, p53 and p21, showing co-localisation of p16, p53 and p21 in the same area, together with some pigment. Melanophages are also seen. The unreactive cells in this area appear to be melanoma cells; many areas of this tumour were unpigmented and negative for all of these markers. (MART1 can be lost in advanced melanomas). Scale bar, 100 *μ*m. Bottom left, p16-positive cells are often large and sometimes have more than one nucleus (arrowheads). Scale bar, 60 *μ*m. Bottom right: nuclear p53 reaction in another RGP melanoma. Again, some positive cells are multinucleate (arrowheads). Scale bar, 30 *μ*m. (**B**) Typical VGP melanoma. Left: rare area of p16 expression. The positive cells (arrows) are large and well-pigmented (melanin, brown). It is unclear whether all pigmented cells have p16, but some have prominent nucleoli (arrowheads). Scale bar, 30 *μ*m. Right: p53 reaction is also rare. Here, the positive cells are again large and colocalised with pigment, and the p53 is cytoplasmic (arrows, cells with clearly negative nuclei). Scale bar, 30 *μ*m. (**C**) CHK2 expression in various lesion types. Top left: benign compound naevus. There is nonspecific reaction and occasional stronger reaction (arrowheads). Top right: dysplastic naevus (same area as in Figure 3A, top). Little reaction is visible. Scale bar, 100 *μ*m. Bottom left: RGP melanoma (same area as in **A**, top). Prominent CHK2 immunostain (arrowheads) colocalises with p16, p53 and p21. Bottom right, VGP melanoma (same area as in **B**), showing prominent specific reaction (arrowheads). Some nonspecific reaction is also seen. Scale bar, 100 *μ*m.

In all primary melanomas examined, we detected neither immunoreactive p21 nor p53 in most cells, although most lesions had a few areas with positive cells (Table 1). Likewise, in many RGP and nearly all VGP melanomas, most areas lacked detectable p16 (Table 1). Even where present, p16 immunoreactivity was generally noticeably fainter and more often cytoplasmic than in naevi (Figure 4A and B). In RGP melanomas, p53 and p21 were often colocalised with each other, with pigmented melanocytic cells, and with p16 (Figure 4A). Melanophages and/or lymphocytes were again often found nearby (Figure 4A). The immunostained cells were often large and sometimes multinucleate (Figure 4A), features of senescent cells as already mentioned. VGP melanomas showed similar colocalisation of the markers studied, typically at the edges of the lesion, with the bulk of the nodule negative. Even in these few positive areas in VGP melanomas, both p16 and p53 tended to be cytoplasmic in location (Figure 4B). In the area shown, some lesional cells were well pigmented and also had prominent heterochromatic foci (Figure 4B).

### Differential CHK2 expression in pigmented lesions

Checkpoint kinase CHK2 is activated and mediates p53 activation on DNA damage and in p53-dependent senescence (d'Adda di Fagagna *et al*, 2003; Von Zglinicki *et al*, 2005). As some activated proteins become stabilised, we surmised that CHK2 levels might change with melanoma progression. Immunostaining for CHK2 partially supported this idea. Most lesions in each category showed some CHK2 reaction, although generally most of each lesion was negative, as with p53 (Figure 4C; Table 1). Intense reaction was observed only in melanomas (Figure 4C; Table 1). CHK2 immunoreactivity was generally present in areas with p53 reactivity (Figure 4C, compare with Figures 2, 3 and 4A). The naevus from the p16-deficient patient was positive for CHK2 (not shown).

### Growth inhibition and p16 elevation by oncogenic *BRAF* in cultured melanocytes

**Figure 5:**
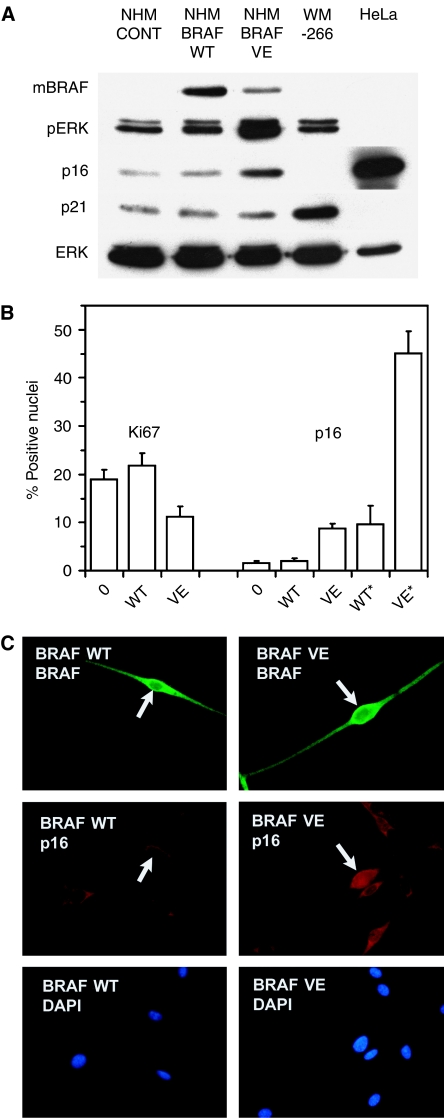
Effects of transfected *BRAF* sequences on cultured normal human melanocytes. Assays were performed 10 days (**A**) or 5 days (**B**, **C**) after transfection of cells. (**A**) Expression of growth-related proteins as shown by immunoblotting. (NHM), normal human melanocytes, transfected with the vector only (CONT, control), or with ^*WT*^*BRAF* (WT) or ^*V600E*^*BRAF* (VE) sequences. WM266-4 human melanoma cells and HeLa cells were used as positive controls for p21 and p16 expression, respectively. mBRAF: detection of Myc tag present on both WT and VE transfected *BRAF* sequences. pERK: phospho-ERK as a marker of active MAPK signalling. ERK: total ERK protein. (**B**) Counts of nuclei positive for proliferative marker Ki67 or for p16, by immunostaining of cells transfected with vector only (0), ^*WT*^*BRAF* or ^*V600E*^*BRAF*. Means and SEM are shown. p16 positivity is shown in both the total culture including any nontransfected cells, and (^*^) as % of double-stained cells positive for exogenous BRAF (Myc tag), that also showed nuclear p16. After only 5 days, Ki67 positivity was significantly reduced in the total culture with ^*V600E*^*BRAF* (*P*<0.05). Nuclear p16 expression was greater in ^*V600E*^*BRAF*-transfected (*P*<0.001) but not ^*WT*^*BRAF*-transfected cells relative to control (whole culture), and in ^*V600E*^BRAF-expressing cells (over 45% positive for p16) relative to ^*WT*^BRAF-expressing cells (*P*<0.001). Significance testing was by Student's *t*-test. (**C**) Typical examples of immunostained cells, showing nuclear p16 staining in melanocytes expressing ^*V600E*^BRAF but not ^*WT*^BRAF. Upper label on each panel indicates transfected sequence, lower label indicates stain. Both BRAF proteins were immunostained with anti-Myc tag. This gave no staining in cultures transfected with the vector only (not shown). DAPI staining showed nuclei.

We and others had speculated that senescence in moles might arise following proliferation due to mitogenic mutations, especially the common ^*V600E*^*BRAF* mutation. Accelerated senescence induced by oncogenes has been reported in other normal human cell types. To investigate this potential mechanism, we tested whether *BRAF* sequences could induce a response resembling accelerated senescence in normal human melanocytes. Cells were transfected with ^*WT*^*BRAF* (wild-type) or ^*V600E*^*BRAF* sequences or a control vector. The Nucleofection procedure gave efficient transfection (typically around 30–40% with *BRAF* sequences), and effects were assessed on the whole, unselected cultures after 5–10 days. Findings were reproduced in four independent experiments, and typical results are shown in Figure 5. As shown in Figure 5A, expression of the *BRAF* sequences could readily be detected. Elevation of MAPK signalling, indicated by ERK phosphorylation relative to ERK abundance, was strong with ^*V600E*^*BRAF* (VE), but not visible with ^*WT*^*BRAF* (WT). Cell proliferation (Ki67 labelling) was significantly reduced in melanocytes transfected with ^*V600E*^*BRAF* but not ^*WT*^*BRAF* (Figure 5B). Note that there was no selection for transfected cells, so the maximum expected growth inhibition is 30–40% (transfection efficiency). Interestingly, expression of p16 was significantly elevated in cultures transfected with ^*V600E*^*BRAF* but not ^*WT*^*BRAF*, especially in individual cells expressing ^*V600E*^BRAF relative to those expressing ^*WT*^BRAF (Figure 5A–C), whereas p21 expression was no higher with ^*V600E*^BRAF than ^*WT*^BRAF or the empty vector (Figure 5A). Immunostaining showed frequent nuclear expression of p16 in individual cells expressing ^*V600E*^BRAF but not ^*WT*^BRAF (Figure 5C). Moreover, further double immunostaining indicated mutual exclusion between nuclear p16 and Ki67 expression among individual cells (data not shown). Thus, the inhibition of melanocyte proliferation by oncogenic but not ^*WT*^BRAF correlates with increased abundance and nuclear location of p16, but not p21.

## DISCUSSION

We report firstly that hTERT expression and p16/RB deficiency are necessary and sufficient for immortalisation of human melanocytes. In agreement with the extended lifespans seen in p16-deficient melanocytes (Bennett and Medrano, 2002), this confirms that cell senescence in cultured human melanocytes is normally p16-dependent. Chudnovsky *et al* (2005) recently described retroviral infection of melanocytes with combinations of genes including mutant CDK4 and hTERT, and reported no short-term alteration in proliferation *in vitro*, but these were recently explanted, presenescent cultures and were not tested for immortalisation. Melanocytes appear unusual in displaying p16-dependent senescence in favourable culture conditions without any apparent stress, unlike p16-dependent senescence in other cell types (Ramirez *et al*, 2001). One possible residual source of ‘stress’ is the supraphysiological oxygen concentration (19%) in 90% air, but Ramirez *et al* made no mention of reducing oxygen tension when they abrogated p16-dependent senescence in keratinocytes and mammary cells using feeder cells, and fibroblasts do not need low oxygen to avoid p16-dependent senescence, so a cell-type difference can still be inferred. This provides a possible rationale for the particular relationship between p16 and melanoma, where germline p16 mutations are predominantly associated with familial melanoma (Hayward, 2003; Kefford *et al*, 2004). Findings from cell culture cannot necessarily be extrapolated to melanocytes in the skin, however, so it was of interest to examine proliferative lesions *in vivo* directly, for possible parallels.

Cell senescence provides an attractive explanation for the biology of moles, which first grow and then stop growing, often remaining static for decades, as we and others have postulated before (Bennett and Medrano, 2002; Mooi and Peeper, 2002; Bastian, 2003; Bennett, 2003). As proposed before (Bennett, 2003; Bennett, 2006), the initial growth stimulus seems likely to be a mitogenic mutation, usually an activating *BRAF* or *NRAS* mutation since these are found in around 80 and 5–15%, respectively, of naevi (Pollock *et al*, 2003; Gray-Schopfer and Bennett, 2006). Here, we report that all predicted molecular and morphological attributes of cell senescence are found in uncultured benign naevi, including expression of p16 and acidic *β*-galactosidase in all tested lesions, markers also recently reported by Michaloglou *et al* (2005). Here, we also report the presence of giant cells, multinucleate cells and heterochromatic foci. From previous publications, naevi show very low expression of proliferative marker Ki67 (Healy *et al*, 1998), and naevus-cell cultures contain large and multinucleate cells and grow poorly (Gilchrest *et al*, 1986; Halaban *et al*, 1986) (reviewed Bennett and Medrano (2002)). p16 expression in melanocytic lesions has been studied previously, with results largely consistent with ours (Talve *et al*, 1997; Keller-Melchior *et al*, 1998). However, the authors did not relate p16 expression to senescence, nor examine other cell-senescence effectors. Expression of p16 in naevi was assumed by these groups to be normal, and was used as a comparison to report downregulation in melanomas. Here however, we find it to be abnormal, since crucially p16 is undetectable in epidermal melanocytes in normal skin, and thus it is overexpressed in naevi. Moreover, p16 expression and growth inhibition could be induced in cultured melanocytes by expression of ^*V600E*^BRAF. This was also recently reported by Michaloglou *et al* (2005), although without any quantitation of p16. They observed growth arrest and acidic *β*-galactosidase in nearly all the cells after 21 days, concluding that the inhibited growth was a form of premature senescence. Here, we additionally report that nuclear translocation of p16 occurred, that ^*WT*^BRAF expression did not impair growth nor increase p16 expression or nuclear location, and that p21 expression was not correlated with the growth inhibition. Thus, if this is senescence, it is not effected by the p53-p21 pathway. It may be either p16-dependent or a novel form of senescence, with as-yet unknown effector(s) in addition to p16. It seems comparable to the premature senescence reported in other cells in response to overexpression of oncogenes such as activated *HRAS*, proposed to be a defensive response to ‘oncogenic stress’ (overstimulation of mitogenic pathways such as the MAPK pathway) (Collado and Serrano, 2006).

The absence of detectable p53 or p21 in most benign naevi, using protocols that did stain these proteins in other lesions, similarly makes it very unlikely that their senescence is p53-dependent. Again, the senescence in naevi may be a novel form of senescence. Otherwise it seems most likely that it is p16-dependent senescence, since most cells in all benign naevi did express p16, and since human melanocytes normally show p16-dependent senescence in culture. It is unclear, however, why p16 cannot be immunostained in all cells of benign naevi, if it mediates their growth arrest. Several reasons seem possible. Firstly, the sensitivity of immunostaining is limited, and the levels of p16 are clearly variable, so it may be that p16 is actually present in all naevus cells and is able to arrest growth at relatively low levels. Secondly, there may be cell–cell interactions such as secretion of growth inhibitors by senescent cells, that secondarily arrest p16-non-expressing cells – and induce senescence marker acidic *β*-galactosidase, which both we and Michaloglou *et al* typically observed in all cells of naevi. Thirdly, this may be an effect of the widespread chromatin remodelling and gene silencing that can result from high activation of RB-family proteins (Narita *et al*, 2003). Cells may become so transcriptionally inactive that p16 itself, although initially expressed, becomes silenced in some cells, growth stasis being maintained by silencing of genes required for cell proliferation.

We proposed previously (Bennett, 2003) that dysplastic naevi might represent escape from p16-dependent senescence and attainment of p53-dependent senescence (or telomeric crisis (Sharpless and DePinho, 2004)). Here, we report that p16 expression is indeed patchy and sometimes cytoplasmic in dysplastic naevi, but generally p53 is expressed only in a few areas, and often without p21. Thus, dysplastic areas of naevi may sometimes be still proliferating rather than in any kind of senescence. We also suggested that primary melanoma cells have emerged from senescence and immortalised (Bennett, 2003). Previous evidence for this includes the obvious property that melanoma cells proliferate, and the detection of telomerase activity in most melanomas and not in naevi (Glaessl *et al*, 1999; Rudolph *et al*, 2000). Explanted melanoma cells are not reported to senesce (Herlyn *et al*, 1985), growing so readily that thousands of melanoma cell lines exist. From immunohistochemistry of parallel sections, we now report that senescence effectors p16, p21, p53 and overexpressed CHK2 are undetectable in substantial regions of all melanomas, while a few areas usually expressed all of these, often in association with large, pigmented and/or multinucleate cells. Such areas may represent residual senescent regions (naevus), part of the clonal evolution of the tumours.

p16 protein at times appeared cytoplasmic-only. This may sometimes indicate a dysfunctional, mutant protein, as it does in the naevus from the p16-mutant patient (Huot *et al*, 2002), although other factors such as other RB pathway alterations may affect p16 location. More commonly p16 was absent from much of a melanoma section, especially in VGP lesions; this can be explained partly by the high rate of p16 deletion in primary melanomas (Curtin *et al*, 2005), but may also involve other mechanisms, since the said deletions are often not homozygous. In some cases, p53-expressing lesional cells in the dermis were surrounded by lymphocytes and melanophages. Among other possible explanations, death of lesional melanocytes may provoke an inflammatory reaction. This is plausible: lesional melanocytes strongly expressing p53 are likely to be p16/RB-deficient, and cultured p16-deficient melanocytes tend to apoptose in the absence of keratinocytes (Bennett, 2003; Sviderskaya *et al*, 2003).

Strong genetic evidence that the proliferative arrest in naevi can be p16-dependent is provided by the frequent association of germline p16 defects with larger, more numerous and dysplastic naevi (Gruis *et al*, 1995; Bennett, 2003), as well as with melanoma susceptibility. Here, we report unusual p53 and p21 expression in a large naevus from a patient who lacks functional p16 and whose cells thus cannot undergo p16-dependent senescence. It is possible that this naevus was undergoing or approaching p53-dependent senescence, although not proven. As the p16 sequence is a melanoma susceptibility gene (Gruis *et al*, 1995; Hayward, 2003), p16 must somehow impede melanoma development. However, not all melanoma families with p16 mutations do have abnormally large naevi (Hayward, 2003; Kefford *et al*, 2004); likewise members bearing the same p16 mutation within a single family can vary as to whether they have large naevi (Gruis *et al*, 1995), which suggests variation between humans in the contribution of p16 compared to other genetic (or environmental) components in determining mole size distribution. This may involve redundancy with some functionally related molecule, as suggested (Gruis *et al*, 1995). The present report together with that of Michaloglou *et al* (2005) provide persuasive evidence that the growth arrest in naevi is cell senescence. This seems to be a highly efficient barrier to melanoma, since naevi are far more common than melanoma. As mediators of p53-dependent senescence are not found in benign naevi, whereas p16 is detected in 100% of these lesions and in most cells within the lesions, it seems likely that p16 at least contributes to establishing senescence in naevi. This provides an attractive possible mechanism by which the p16/RB pathway could suppress melanoma following mitogenic mutations like *BRAF* activation. It was reported recently that RB pathway ablation in combination with three other genetic alterations in human melanocytes (*NRAS* activation, p53 activation and hTERT expression) is necessary and sufficient to produce melanoma-like lesions in a reconstituted skin-xenograft model (Chudnovsky *et al*, 2005), in good agreement with our genetic model (Bennett, 2003).

There is much potential for further exploration of the possibility of cell senescence *in vivo* and as a real cancer barrier. Cancers grow progressively, but various lesions such as cysts and thyroid adenomas show self-limiting growth. Perhaps cell senescence may occur in many organ types following initial mutations, as supported by recent reports from animal models (Braig *et al*, 2005; Collado *et al*, 2005). This would help to explain why p16 and p53 are such universal tumour suppressors, and it now seems important to investigate this possibility more widely.

## Figures and Tables

**Table 1:** Differential expression of p16, p53, p21 and CHK2 in pigmented lesions of increasing malignancy

	**Proportion of lesions positive**											
	**p16**			**p53**			**p21**			**CHK2**		
**Lesion type**	**++^a^**	**+^a^**	**All^a^**	**++**	+	**All**	**++**	+	**All**	**++**	+	**All**
Benign compound naevi	**10**/**11**	1/11	11/11	0/7	1/7	**1/7**	0/9	**0/9**	**0/9**	0/8	6/8	6/8
Dysplastic naevi	**5/9^b^**	4/9	9/9	0/7	4/7	**4/7**	0/7	**2/7**	**2/7**	1/6	3/6	4/6
RGP melanomas	**7/14^b^**	7/14	14/14	0/8	6/8	**6/8**	0/6	**6/6**	**6/6**	2/7^c^	5/7^c^	7/7^c^
VGP melanomas	**3/14b,^d^**	11/14	14/14	1/8	7/8	**8/8**	0/8	**4/8**	**4/8**	0/8	6/8^c^	6/8^c^

a ++: fraction of lesions that were predominantly positive (i.e. with 51–100% of cells immunostained), out of number of lesions assessed. +: fraction of lesions with detectable immunostaining up to 50% of cells). All: all positive lesions, sum of + and ++. Lesions were scored (0, +, ++) by two independent observers, who agreed on the scores.

b Some cytoplasmic-only reaction. Reaction generally appeared fainter in melanomas than in naevi.

c Some lesions with intensely positive areas.

d VGP melanomas that were predominantly p16-positive were all relatively small. Columns in bold show a trend between lesion types (*P*<0.01, *P*<0.02, *P*<0.005, respectively, for p16, p53 and p21, by the *χ*^2^ test).
